# Phyto-synthesized silver nanoparticles from *Sargassum subrepandum*: anticancer, antimicrobial, and molluscicidal activities

**DOI:** 10.3389/fpls.2024.1403753

**Published:** 2024-05-08

**Authors:** Heba El-Sayed, Asmaa Abdelsalam, Mostafa Y. Morad, Hana Sonbol, Amina M. Ibrahim, Eman Tawfik

**Affiliations:** ^1^ Botany and Microbiology Department, Faculty of Science, Helwan University, Helwan, Egypt; ^2^ Zoology and Entomology Department, Faculty of Science, Helwan University, Helwan, Egypt; ^3^ Department of Biology, College of Science, Princess Nourah Bint Abdulrahman University, Riyadh, Saudi Arabia; ^4^ Medical Malacology Department, Theodor Bilharz Research Institute, Giza, Egypt

**Keywords:** Anticancer, *Biomphlaria alexandrina*, macroalagae, breast cancer, *Sargassum subrepandum*, *in silico*, antimicrobial

## Abstract

In the realm of nanotechnology, the use of algae to produce nanoparticles is an environmentally friendly, sustainable, and economically viable strategy. In the present study, the brown macroalgae *Sargassum subrepandum* was utilized to effectively produce silver nanoparticles (AgNPs). Through various characterization techniques, the AgNPs’ structural integrity was confirmed. AgNPs exhibited significant antimicrobial activity against *Pseudomonas aeruginosa* and *Fusarium equiseti*. AgNPs showed cytotoxic effects on the MCF-7 breast adenocarcinoma cell line with an IC_50_ of 12.5 µg/ml. Treatment with AgNPs resulted in a marked reduction in cell viability, alongside evident apoptotic and necrotic morphological changes in the cancer cells. Through molecular docking studies, a deeper understanding of the interaction between AgNPs and crucial proteins related to cancer has been achieved, AgNPs showed a promising molluscicidal action on *Biomphalaria alexandrina* snails, a *Schistosoma mansoni* intermediate host. The half-lethal dose (LC_50_) of AgNPs was determined to be 0.84 mg/L. The potential consequences of its administration include potential disruptions to the glycolysis profile, as well as potential impacts on the steroidal hormone’s estrogen and testosterone and certain kidney function tests. This study highlights the diverse uses of algae-synthesized AgNPs, ranging from healthcare to environmental management, demonstrating their importance in advancing nano-biotechnological solutions.

## 1 Introduction

In recent years, nanotechnology has emerged as a novel and distinct technological field, offering a diverse array of applications in the domains of medicine, agriculture, and industry (Malik et al., 2023). The green biosynthesized nanoparticles have gained great attention for their safe biological activities, as these particles possess promising characteristics and find vast applications in several domains. Moreover, their eco-friendly nature further enhances their appeal (Nasrollahzadeh et al., 2019).

Bio fabricated nanoparticles have shown great potential as a viable option to replace conventional chemical antibiotics in the fight against microbial infections, particularly in the field of medicine. Recent studies have brought attention to different methods and advantages of utilizing biomaterials as effective antimicrobial agents. For example, Allawi (2024) explored the potential of using biosynthesized gold nanoparticles from clove extracts to treat and prevent oral biofilms caused by oral bacteria. Abdelbaky et al. (2022), documented the antibacterial, antioxidant and anti-inflammatory effectiveness of zinc oxide nanoparticles synthesized via *Pelargonium odoratissimum* aqueous leaf extract. AgNPs synthesized from the leaves of *Gardenia thailandica* exhibited antibacterial properties against *Staphylococcus aureus* (Attallah et al., 2022). AgNPs produced from cyanobacteria have been reported to be effective antibiofilm agents against *Candida albicans* (Ahamad et al., 2021). Moreover, the antioxidant and antibacterial properties of AgNPs synthesized using *Caulerpa sertularioides* algae were also reported (Anjali et al., 2022).

In the realm of cancer therapy, the utilization of biosynthesized nanoparticles as anticancer agents represents a frontier in the development of more targeted and less toxic cancer therapies (Gavas et al., 2021). Biosynthesis of nanoparticles involves eco-friendly processes using biological entities like plants, bacteria, fungi, and algae. This method not only offers a green alternative to traditional chemical and physical methods but also imbues the nanoparticles with unique biological properties beneficial for cancer treatment (Ovais et al., 2021). Moreover, the biocompatibility and reduced toxicity of biosynthesized nanoparticles make them an attractive alternative to traditional cancer therapies (El-Sheekh et al., 2023). Furthermore, biosynthesized nanoparticles possess the capacity to be modified with ligands, enabling them to target cancer cells more effectively (Karmous et al., 2020). It can be designed to encapsulate cancer-fighting drugs, so improving their ability to dissolve and be absorbed by the body (Wei et al., 2019; Singh et al., 2022). Ongoing research is focused on optimizing the synthesis processes, improving the targeting accuracy, and understanding the interaction between these nanoparticles and biological systems at the molecular level. As more studies uncover the full potential of these nanoparticles, they are poised to play a significant role in the future of cancer treatment.

Connecting the huge range of nanotechnology applications with global health challenges, millions of people and animals suffer from schistosomiasis, a parasitic disease requiring effective control measures (Morad et al., 2023). In 2019, about 236.6 million people needed treatment for schistosomiasis, according to a World Health Organization report released in January 2022 (https://www.who.int/news-room/fact-sheets/detail/schistosomiasis). The parasitic trematode species known as *Schistosoma mansoni*, which is found in various African and South American nations, is the main cause of schistosomiasis. The *Biomphalaria alexandrina*, a freshwater snail belonging to the Phylum Mollusca and Class Gastropoda, serves as the parasite’s intermediate host. The biological methods can be more effective, safer, and less expensive than chemical ways in managing snail populations. The chemical methods have a few drawbacks such as costly expenses, toxicity towards non-target organisms, and polluting the environment (Ibrahim and Ghoname, 2018). Nanoparticles that are biosynthesized tend to be more biodegradable compared to those that are chemically synthesized, resulting in a reduced long-term environmental impact (Ayilara et al., 2024).

Marine resources are currently undergoing thorough investigation as potential candidates for antibacterial and anticancer medications because they have few adverse effects. Marine algae are regarded as valuable sustainable marine supplies, and their ability to produce NPs has attracted attention. Algal biosynthesized nanoparticles are preferred over conventional methods due to their cost-effectiveness, and the ability to easily scale up production (González-Ballesteros and Rodríguez-Argüelles, 2020). They can be engineered to carry drugs, genes, or proteins directly to cancer cells, minimizing the adverse effects on healthy cells and improving the therapeutic outcomes (Chaudhary et al., 2020). The versatility of algae, with their diverse species offering a wide range of metabolic capabilities, further enhances the potential of this approach. In addition, algae include various organic compounds such as pigments, proteins, enzymes, polysaccharides, vitamins, carbohydrates, and secondary metabolites. These compounds have the potential to operate as natural reducing molecules in the production of nanoparticles (Thiurunavukkarau et al., 2022).

*Sargassum*, a brown macroalga, belongs to the family Sargassaceae, with a vast distribution in many marine habitats globally (Ody et al., 2019). Chemically, it exhibits a high concentration of various biologically active metabolites such as sterols, terpenoids, and phenols (Rushdi et al., 2020). These compounds exhibit a variety of pharmacological effects, including antioxidant properties, anti-cancer activity, immunomodulatory, osteogenic, hypoglycemic, anticoagulant, and antimicrobial effects (Rushdi et al., 2020; Zhang et al., 2020). For example, phlorotannin’s, a class of polyphenolic chemicals identified in brown algae, have been documented to induce apoptosis and impede the growth of cancer cells. Zheng et al. (2022) conducted a study that found that phlorotannins derived from the brown seaweed *Ecklonia cava* have a notable ability to kill leukemia cells by inducing apoptosis. Fucoxanthin, a carotenoid found mainly in brown macroalgae, has been acknowledged for its anti-cancer characteristics, particularly its capacity to cause cell cycle arrest and death. Ahmed et al. (2022) research provides evidence that fucoxanthin can cause G1 cell-cycle arrest in human cancer cell lines, suggesting its potential as an anticancer drug. These chemicals can be used as reducing agents, stabilizing agents, or capping agents during the process of nanoparticle formation (Chugh et al., 2021).

The objective of this study is to utilize *Sargassum subrepandum* for the environmentally friendly production of silver nanoparticles (AgNPs) and to thoroughly assess their biological activities against microbes, cancer cells, and *Biomphalaria alexandrina* snails.

## 2 Materials and methods

### 2.1 Algal collection and identification

**Figure 1:**
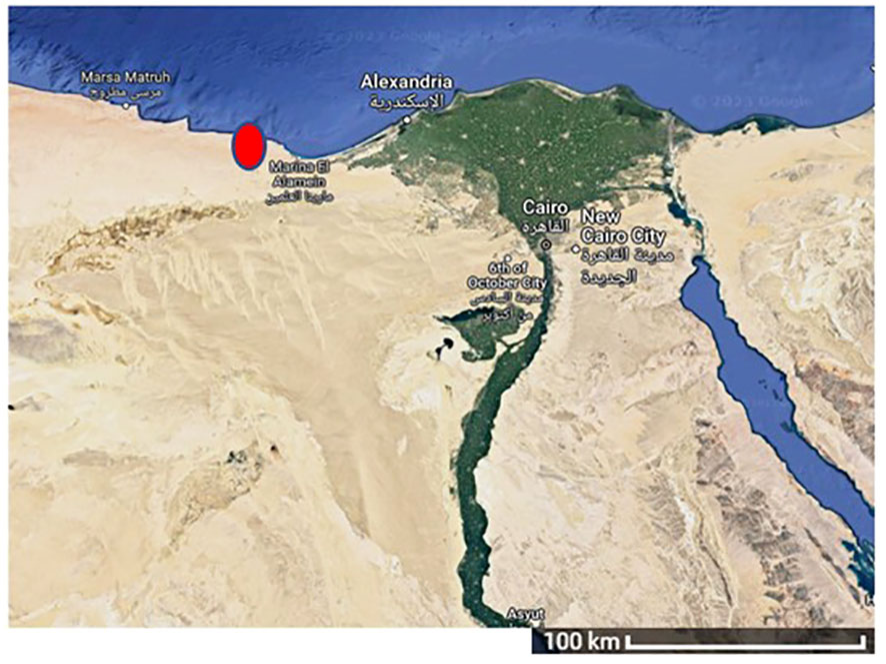
Egypt map showing the site of *S. subrepandum* collection.

The healthy and fresh *Sargassum subrepandum* (*S. subrepandum*) macroalgae were collected from the Northern Coast of Egypt at coordinates 28° 2′ 55.7′ 11″ N, 34° 26′ 13.7′ 28″ E (**Figure 1**). The collected algal samples were stored in polythene bags. They were then transported to the Genetic and Molecular Biology lab, Faculty of Science, Helwan University, Egypt under chilled conditions to maintain temperatures between 4-8°C.

The algae was previously identified, according to Soliman and Tawfik (Soliman and Tawfik, 2021), by the 18S rRNA gene as *Sargassum subrepandum* var. *rueppellii* by a homologous identity of 99% and deposited in Genbank with the accesssion number CH004439.

### 2.2 Extract preparation

The algal samples underwent a thorough rinsing process using sterile-filtered seawater. The thallus part was then washed with tap water for 5 minutes, followed by three additional washes with dH_2_O, each lasting 5 minutes.

After being dried in the shade, the material is then cut into small pieces and ground into a powder using a mixer grinder. Next, 0.25 g of dry powder was extracted using 25 ml of high-grade ethanol according to (Eltak et al., 2023). The mixture underwent agitation at a speed of 130 rpm for a duration of 12 hours, maintaining a temperature of 25°C. Next, the extract was filtered using Whatman paper No. 1 until a clear supernatant was obtained. The extract was stored at a temperature of 4°C for future use.

### 2.3 Biofabrication of silver nanoparticles

The phyco-synthesis of AgNPs was conducted employing the methodology described in (Kumar et al., 2022). Briefly, 1 ml of 100 mM AgNO_3_ aqueous solution (Biotech, 05742) was combined with 9 ml of *S. subrepandum* extract and the mixture was incubated at 25°C in the dark for 24 h. The purification of AgNPs was accomplished by centrifuging the solution mixture at 5600 xg for 30 minutes. Following that, the resulting nano pellet was washed three times with sterile double distilled water, followed by one wash with ethyl alcohol to remove any contamination. Finally, the pellet was vacuum dried to obtain dry powder of AgNPs.

### 2.4 AgNPs characterization

#### 2.4.1 Visual characterization

The initial verification of the biogenesis of silver nanoparticles was first performed by the observable alteration in color of the mixture from green to brown.

#### 2.4.2 UV-Visible spectroscopy

The confirmation of the synthesis of AgNPs was also achieved through the observation of their kinetic behavior using a JASCO V-630 UV-visible spectrophotometer (serial number: C285061148) using a wavelength range of 200-900 nm. The data was collected and analyzed using the Spectra Measurement software.

#### 2.4.3 Transmission electron microscope of AgNPs

The shape and size of AgNPs were assessed through the utilization of TEM imaging tools. To prepare the samples, carbon-coated 400 mesh copper grids were positioned over a single droplet of the nanoparticle solution,allowing them to incubate for a duration of 1.5 minutes. Subsequently, a single droplet of 2% uranyl acetate filtared solution was added to the grid for an additional 1.5 minutes. The extra uranyl acetate was carefully eliminated using filter paper (Kiang et al., 2004). The grids were subjected to a 10-minute period of drying and thereafter analyzed with an electron microscope situated at the Regional Center for Mycology and Biotechnology, Al-Azhar University.

#### 2.4.4 Zeta size and potential measurements

The measurment of zeta size and potential for the investigated silver nanoparticles was conducted using a Zetasizer Nano ZS 7.12 instrument (Zetasizer Nano ZN, Malvern Panalytical Ltd., Malvern, UK) as part of this study. The assessment of particle size was performed via non-invasive backscatter within cuvettes, with measurements being conducted at a temperature of 37°C. Dynamic light scattering was employed to capture the scattered light at an angular orientation of 173°. To mitigate the potential issues arising from multiple scattering phenomena, a sample volume of 1 mL was utilized, and prior to analysis, dilutions were carried out at a ratio of 1:10, comprising 900 µL of buffer and 100 µL of the nanoparticle samples. In the case of chitosan suspensions, the buffer employed was a 50 mM acetic acid solution adjusted to a pH of 3.0. The effective hydrodynamic diameter of the nanoparticles was calculated utilizing the diffusion coefficient derived from the Stokes-Einstein equation, employing the cumulants method. Zeta potential measurements were also conducted concurrently at 37°C, utilizing both types of phase analysis light scattering and laser Doppler velocimetry, known as PALS. The electrophoretic mobility values measured were subsequently translated into zeta potential values utilizing the Smoluchowski approximation. To ensure accuracy and reliability, the zeta potential and hydrodynamic diameter values were expressed as means, accompanied by their respective standard deviations. These values were derived from three independent measurements.

#### 2.4.5 Fourier transform infrared analysis

The FTIR absorption spectra was examined using FTIR spectroscopy (PerkinElmer spectrometer, Akron, OH, USA) to assess the potential interactions between the synthesized AgNPs and the metabolites derived from the algal extract. In this experiment, a small amount of nanoparticles underwent grinding with potassium bromide to produce a compacted pellet. The experiment was conducted within the spectral range of 400 to 4000 nm.

### 2.5 Biological activity of AgNPs

#### 2.5.1 Antimicrobial activity

##### 2.5.1.1 Microbial strains and growth conditions

To investigate the antimicrobial activity and genetic stability impact of AgNPs on microorganisms, two pathogenic microorganisms, namely *Pseudomonas aeruginosa* and the fungus *Fusarium equiseti*, were chosen for this study.

*P. aeruginosa* was generously supplied by the Agriculture Centre of Genetic Engineering and Biotechnology, Faculty of Agriculture, Ain Shams University (ACGEB). The strain was morphologically and biochemically characterized in accordance with the guidelines outlined in Bergey’s manual. A single colony of *P. aeruginosa* was inoculated into Luria-Bertani (LB) agar medium, consisting of 5 g of yeast extract, 10 g of tryptone, 10 g of NaCl, 20 g of agar, and 1 ml of 1N NaOH, with the pH adjusted to 7.4. The bacterial culture was incubated at a temperature of 37°C for 24 h (Maniatis et al., 1983).

*F. equiseti* (Link), was obtained from the Genetic and Molecular Biology Lab at Helwan University’s Faculty of Science. It was identified by Sigma Scientific Services Co. using NRRL 26419 ITS region from the TYPE material. *F. equiseti* mycelia were cultivated on Petri dishes supplemented with potato dextrose agar (PDA), composed of 200 g of potato, 20 g of dextrose, and 20 g of agar. The fungal culture was cultured for a duration of 7 days at a temperature of 28°C.

##### 2.5.1.2 Antibacterial activity of AgNPs against *P. aeruginosa*

The Agar Well Diffusion Assay was employed to assess the antibacterial efficacy of AgNPs. Briefly, 100 µl of suspension culture (1×10^6^ CFU/mL) was added to 20 mL of LB agar medium, ensuring equal dispersion. A cork borer with a diameter of 6 mm was utilized to generate wells in the agar plates. Subsequently, a volume of 100 µl of silver nanoparticles at a concentration of 0.1M was added to each well. The positive control in this experiment involved the use of Gentamycin (10 µg/disc), while the negative control consisted of a well containing 100 µl of distilled H_2_O. The presence of inhibitory zones surrounding the wells indicated the antibacterial efficacy of the nanoparticles. Subsequently, the measurement of the inhibitory zones was conducted in mm. Three replicates were created for each treatment (Reeves and O, 2003).

##### 2.5.1.3 Antifungal activity of AgNPs against *F. equiseti*

The antifungal efficacy of AgNPs was evaluated by measuring the inhibition of mycelial growth of *F. equiseti*. Briefly, it was cultivated in Potato Dextrose Agar (PDA) medium at a temperature of 25°C for a duration of five days. The method selected to evaluate the antifungal activity was the percent inhibition of mycelial growth (PIMG) technique (Hamad et al., 2022). The plates were inoculated with a 100 µl volume of AgNPs at a concentration of 0.1M. PDA Fungal plugs of 5 mm in diameter were immediately injected into the central region of the medium. Negative control agar plugs with the identical diameter as those used were inoculated into the sterile potato dextrose agar (PDA) medium. The cultures were incubated for seven days at a temperature of 28°C. Following the incubation period, the diameter of fungal plates treated with AgNPs was measured and compared to a control group.

The formula for calculating the percentage increase in diameter (PIMG) is derived as follows:


PIMG=Gc−Gt/Gc×100

Where, Gc represents the growth diameter seen in the control treatment. The growth diameter on plates treated with AgNPs is denoted as GT.

##### 2.5.1.4 Gene toxicity of the AgNPs treated microorganisms

a.DNA isolation.

The genomic DNA from *P. aeruginosa* pellets and *F. equiseti* mat was extracted via the cetyltrimethylammonium bromide (CTAB) approach, as described by Edwards et al. (year). The DNA quality and concentration was conducted using the NanoDrop-1000^®^ Spectrophotometer, manufactured by Nano-Drop Technologies in Wilmington, DE, USA.

b. Inter Simple Sequence Repeat (ISSR) assay.

The Inter Simple Sequence Repeat (ISSR) approach was utilized to assess the impact of AgNPs on the gene stability of *P. aeruginosa* and *F. equiseti*.

The PCR reaction was conducted using the following components: 25 µl of the Red Taq master mix (Biolene), 12.5 µl of genomic DNA, 1 µl of each primer (Biosearch, #P 1–5), and 9.5 µl of ddH_2_O. The reaction program consisted of 35 cycles, which were executed according to the following instructions. The denaturation step was carried out for a duration of 30 seconds at a temperature of 94°C. Subsequently, the annealing step was performed at a specified temperature for 30 seconds for each primer. Finally, the extension step was conducted for a period of 1 minute at a temperature of 72°C. Subsequently, a final extension phase was conducted by subjecting the sample to a temperature of 72°C for a duration of 10 minutes, followed by a subsequent cooling to a temperature of 4°C.

The analysis of the PCR outcomes involved the utilization of a 1.5% agarose gel, which was afterwards stained with ethidium bromide (Sigma). The bands that resulted from the experiment were visualized and captured using the Gel Doc XR+ imager^®^ manufactured by Bio-Rad, located in Hercules, California, USA. The determination of band sizes was facilitated through the utilizations of a 1kb plus DNA ladder (New England Biolab, #N3232S).

#### 2.5.2 Anticancer activity of AgNPs

##### 2.5.2.1 Cell culture

The MCF-7 breast adenocarcinoma cell line, was acquired from Nawah Scientific Inc. lab, located in Mokatam, Cairo, Egypt. The cells were cultured in Dulbecco’s Modified Eagle Medium (DMEM) included streptomycin at a concentration of 100 mg/mL, penicillin at a concentration of 100 units/mL, and heat-inactivated fetal bovine serum at a concentration of 10%. The culture was maintained in a humidified environment with a 5% (v/v) concentration of carbon dioxide (CO_2_) at a temperature of 37°C (Hamad et al., 2022).

##### 2.5.2.2 Cytotoxicity assay

The cytotoxicity of AgNPs was investigated by employing the Sulforhodamine B (SRB) assay, as stated in (Skehan et al., 1990). 100 µL of a cell suspension containing 5x10^3^ cells was evenly divided over 96-well plates and incubated in the medium for 24 hours. Subsequently, the cells were treated with a 100 µL of the medium with various doses of AgNPs (varying from 0.01 to 100 μg/ml) for a period of 72 hours. Then the media was modified to include 150 µL of a 10% solution of trichloroacetic acid (TCA) and incubated at a temperature of 4°C for 1 hour to induce cell fixation. Subsequently, the TCA solution was disposed of, and the cells underwent a thorough rinsing process with double-distilled water, repeated five times, to achieve comprehensive cleansing. The cells were cultured for 10 minutes at room temperature in a totally light-deprived environment. During this time, 70 µL of SRB solution (0.4% w/v acetic acid) were introduced to the plates to do three rounds of washing. Subsequently, the plates were left overnight to allow for drying. Subsequently, a volume of 150 μL of TRIS solution with a concentration of 10 mM was added, and the absorbance at a wavelength of 540 nm was determined utilizing a BMGLABTEH^®^-FLUOstar Omega microplate reader (Ortenberg, Germany). The experiment was conducted using three replicates.

##### 2.5.2.3 Flow cytometry assay

The determination of apoptosis and necrosis cell populations was conducted with the Annexin V-FITC apoptosis detection kit (Abcam Inc., Cambridge Science Park, Cambridge, UK) in conjunction with a flow cytometry system equipped with two fluorescent channels. Following the application of AgNPs (12.5 µg/ml) for 24 h, during the specified time frame, a total of 10^5^ cells were gathered by the process of trypsinization. The cells were then rinsed twice with ice-cold PBS solution at a pH of 7.4. Subsequently, the cells are subjected to incubation in a light-deprived environment with a volume of 0.5 ml of Annexin V-FITC/PI solution for a duration of 30 minutes at room temperature, in accordance with the guidelines provided by the manufacturer. Following the staining process, the cells were inserted into the ACEA Novocyte™ flow cytometer (ACEA Biosciences Inc., San Diego, CA, USA) and subsequently examined for FITC and PI fluorescent signals using the FL1 and FL2 signal detectors, respectively. The excitation/emission wavelengths used were 488/530 nm for FITC and 535/617 nm for PI. In each instance, a total of 12,000 events are recorded and the number of FITC and/or PI cells that exhibit positive characteristics is determined using quadrant analysis. The quantification process is facilitated by the utilization of ACEA NovoExpress™ software, developed by ACEA Biosciences Inc., located in San Diego, CA, USA.

##### 2.5.2.4 *In silico* study

The molecular docking investigation was carried out to predict the interaction between the ligand, AgNPs, and certain breast cell cancer proteins, including survivin, JAK2, and BRCA 2 proteins. The structures of selected proteins were retrieved from the Protein Data Bank (survivin PDB ID 1E31; JAK2 PDB ID 3KRR; BRCA2 PDB ID 3EU7). The molecular docking calculations were performed using the Molecular Operating Environment programme (MOE 2014.09). The molecular structure of Ag atoms was generated using Chem Bio office drawing programme, followed by energy minimization, as described in Wasukan et al (Wasukan et al., 2019). All unattached water molecules and atoms were eliminated. Then, polar hydrogen atoms were added to the chosen proteins, and partial charges determined.

#### 2.5.3 Molluscicidal activity of the biosynthesized AgNPs

##### 2.5.3.1 Snails

Eight to ten millimeter-diameter *B. alexandrina* (Ehrenberg, 1831) snails were acclimated in the Medical Malacology Laboratory of Theodor Bilharz Research Institute (TBRI), Giza, Egypt. The feeding of the snails included oven-dried lettuce leaves, blue-green algae (Nostoc muscorum), and tetramin in plastic aquaria (16 x 23 x 9 cm). The experiment’s water conditions included dechlorinated aerated tap water (10 snails/L), pH of 7.0 and temperature of 25°C, in glass plates.

##### 2.5.3.2 Molluscicidal activity assay

Briefly, the stock solution of AgNPs was used to prepare different concentrations (20, 15, 10, 5, 2.5, and 1 mg/L). For each concentration, ten snails were used with three replicates, then exposed to AgNPs for 96-hours and subsequently by a 24-hour recovery period. For the control group (30 snails), only dechlorinated water was added. The LC_90_ and mortality rate were calculated according to (Morad et al., 2022).

##### 2.5.3.3 Biochemical effects of AgNPs on B. alexandrina snails

###### 2.5.3.3.1 Experimental design

In each tank, ten snails were treated for 96 hours (exposure) to sub-lethal concentrations of AgNPs at LC_10_ (0.11 mg/l) or LC_25_ (0.45 mg/l), followed by another 24 hours (recovery), two weeks of repetition, and two weeks of recovery.

###### 2.5.3.3.21 Tissue preparation

The snails’ shells were removed, and the fleshy components were weighted (1g tissue/10ml phosphate buffer), and then blended in a glass Dounce homogenizer. The tissue homogenates were subjected to centrifugation at a speed of 3,000 rpmper minute for a duration of 10 minutes. Subsequently, the resultant liquid portion (supernatant) was preserved at a temperature of -80°C for biochemical analysis.

###### 2.5.3.3.3 Determination of fluctuations in glycogenolysis and lipid profiles

Using a colorimetric method described in (Trinder, 1969), glucose was measured. Succinate dehydrogenase (SDH) leve was determined using a colorimetric method based on (Rice and Shelton, 1957). Triglycerides level was determined colorimetrically according to (Pande et al., 1963; Beers et al., 1995). Phospholipids (PLs): it was detected colorimetrically according to (Otles and Ozgoz, 2014). The quantification of creatinine concentrations was performed using the colorimetric technique stated by (Tanganelli et al., 1982). The enzymatic procedure, as described in (Barr, 1990), was utilized for measuring uric acid.

###### 2.5.3.3.4 Determination of testosterone and estradiol hormones concentrations:

The hermaphrodite gland tissues of ten snails were employed. Testosterone and estradiol hormone levels were measured for all groups according to manufacturers instructions of the testosterone EIA kit (Enzo Life Science, Michigan, USA, ADI-900-065) and the estradiol EIA kit (Cayman Chemical Company, Michigan, USA, item no. 582251). Briefly, using fresh, disposable tips, 25 µl of each standard, control, and treated samples were dispensed into the proper wells of a microplate coated with either T or E monoclonal antibody. The 200 µl enzyme conjugate was then added to each microplate well, carefully mixed, and allowed to sit at room temperature for 60 minutes. The microplates were then processed, and absorbance was measured (Gholib et al., 2019).

### 2.6 Statistical analysis

Probit analysis (Finney, 1971) was used to analyze the data using SPSS 17.0 for Windows (SPSS Inc. 2008). The data were presented as mean ± S.D., and the student’s t-test was used to compare two means.

Images produced by the gel electrophoresis were evaluated. A band’s presence was given a score of 1, while its absence was given a score of 0. The Jaccard’s similarity coefficient was used to create a pairwise similarity matrix. Cluster analysis was done to create a dendrogram using the unweighted pair group technique with the arithmetic averaging algorithm (UPGMA). Bio-Rad Quantity One was used for these computations (4.6.2). Software from the Community Analysis Package (1.2) was used to obtain the overall dendrogram for all primers (Shuaib et al., 2007). Using the mean, the average, and the standard deviation from the data collected, the analysis of variance test in SPSS 21 was performed (SD) (p ≤ 0.05).

## 3 Results

### 3.1 Nanoparticles characterization

#### 3.1.1 Visual observation

The fabrication of silver nanoparticles from the ethanol extract of *S. subrepandum* was initially observed through the visible colour change of the algal extract and AgNO_3_ mixture from green to brown.

#### 3.1.2 UV-Visible spectroscopy

**Figure 2:**
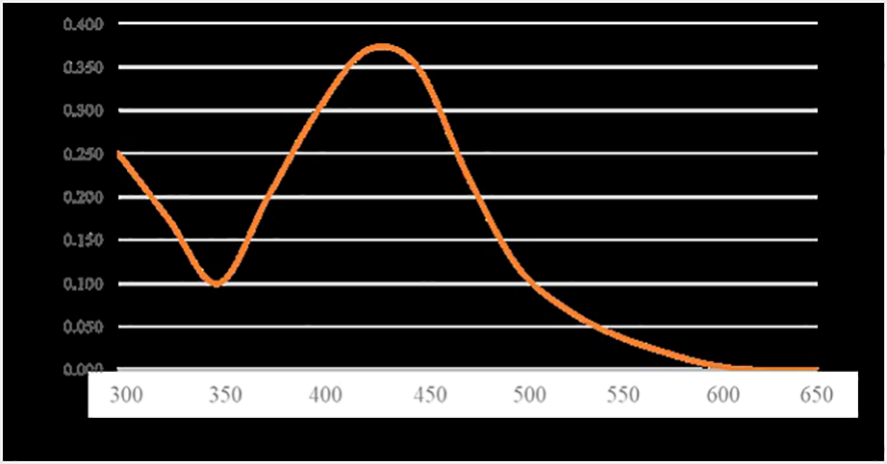
UV-visible measurements of AgNPs.

The ultraviolet-visible (UV-Vis) spectra of AgNPs are shown in **Figure 2**. The absorption peak of AgNPs synthesized using *S. subrepandum* was observed at a wavelength of 425 nm.

#### 3.1.3 Transmission electron microscope

**Figure 3:**
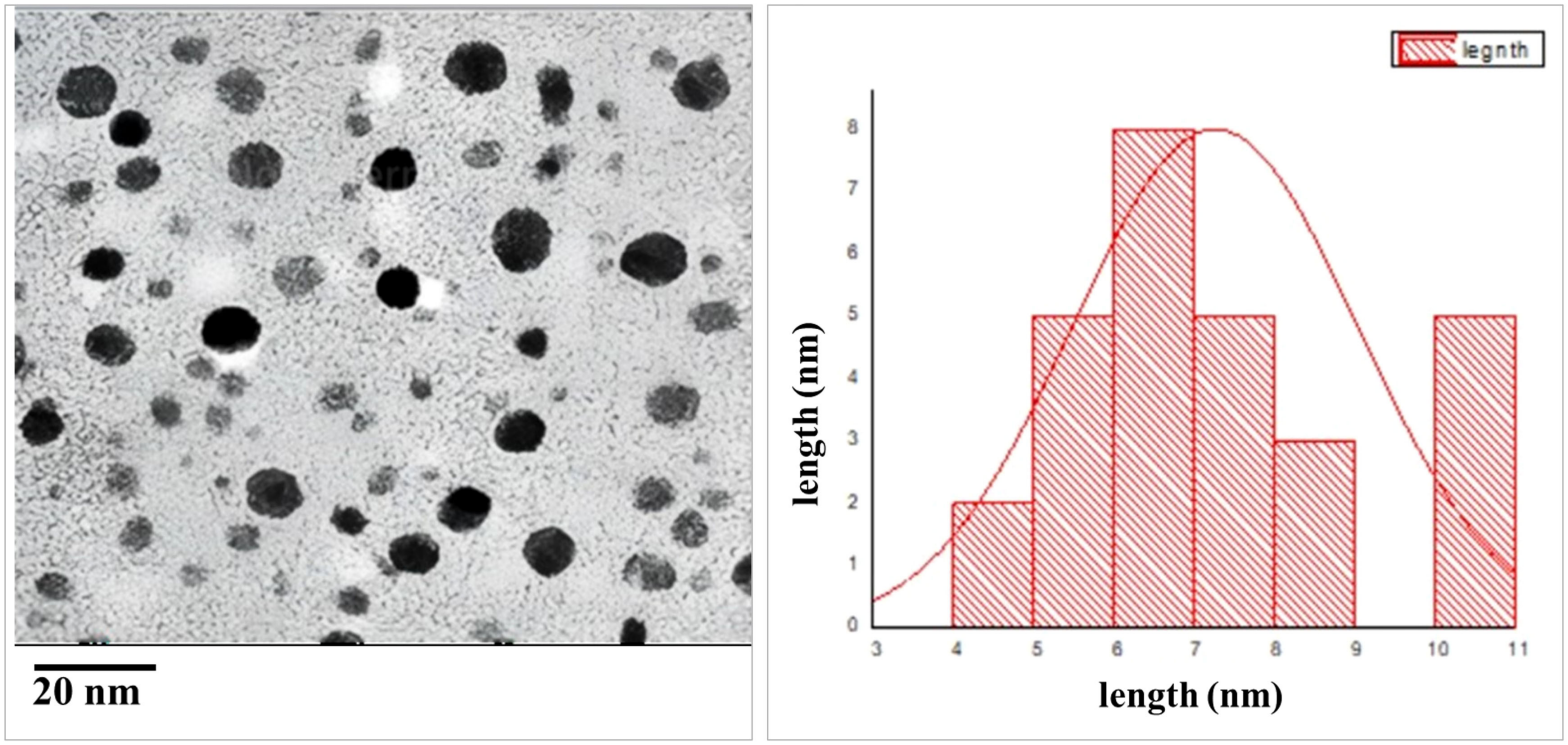
TEM images of AgNPs size. **(A)** Particle size at scale bar 20 nm, **(B)** particle size distribution.

TEM was employed to visualize and measure the shape, distribution, and size of the phycosynthesized AgNPs, as shown in **Figure 3**. TEM analysis of AgNPs revealed that they exhibited characteristics of spherical to cube in shape with a uniform distribution and minimal aggregation. The average particle size was determined to be 4.5 ± 1.2 nm.

#### 3.1.4 Particle size and zeta potential

**Figure 4:**
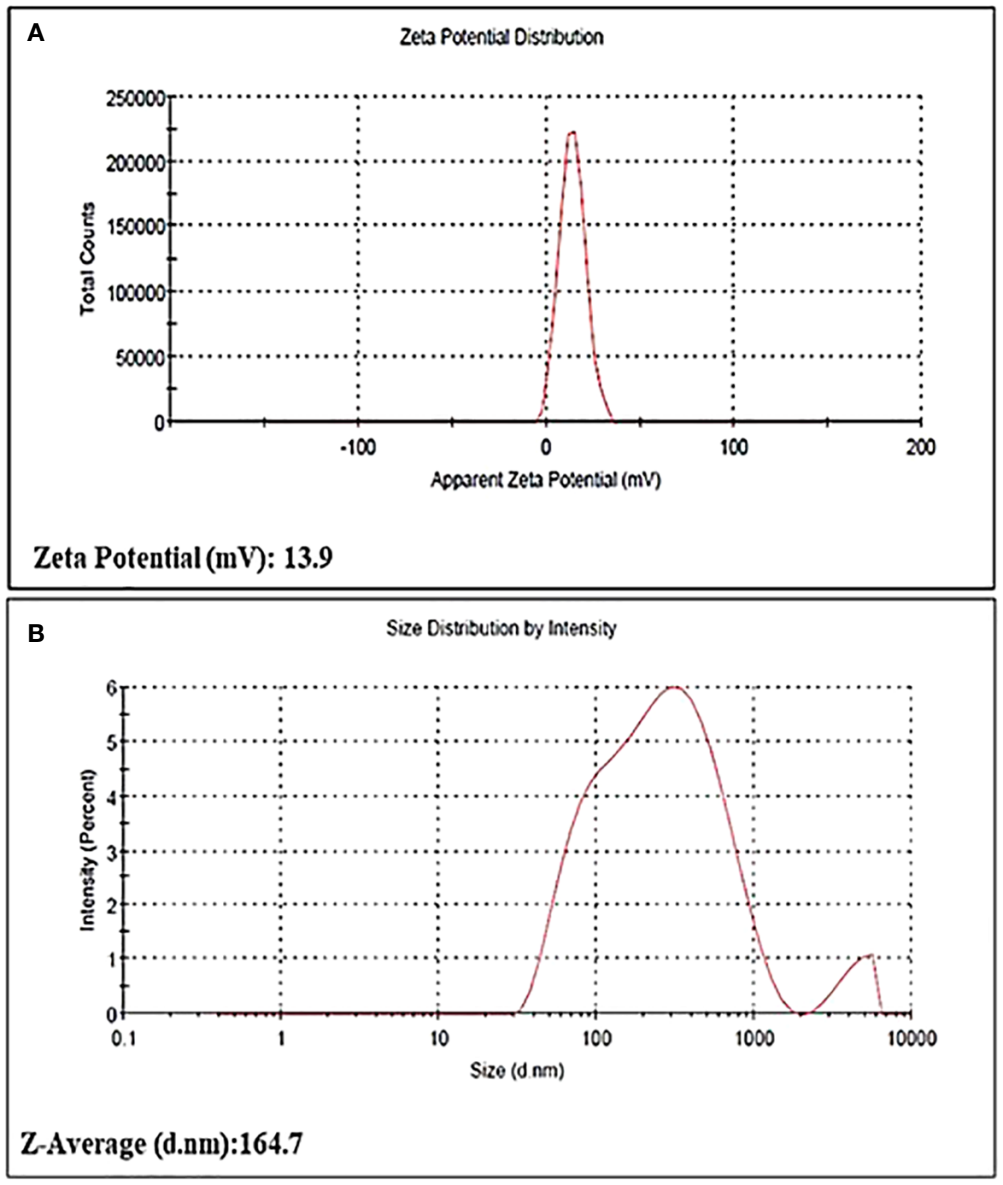
**(A)** Zeta potential distribution, **(B)** Particle size distribution of AgNPs.

The particle size of the bio-fabricated AgNPs was determined using a zeta sizer (Malvern) and found to be 164.7 nm. AgNPs demonstrated notable stability, as evidenced by a zeta poten tial measurement of 13.9 ± 0.37 mv (**Figure 4**).

#### 3.1.5 FTIR analysis

**Figure 5:**
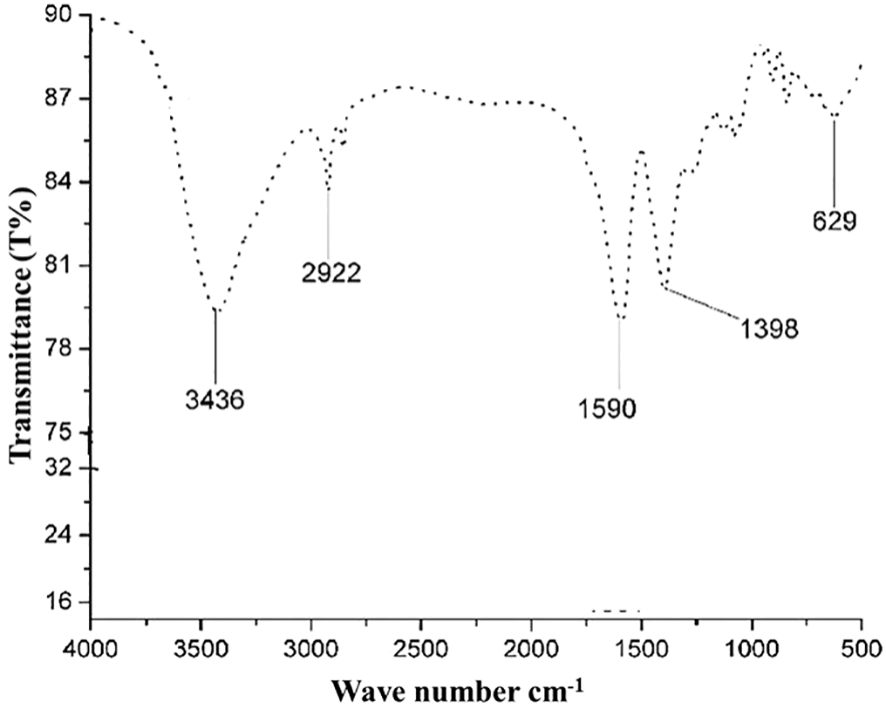
FTIR spectrum of biosynthesized AgNPs.

The FTIR spectrum verified the potential interaction between silver nanoparticles and capping agents in *S. subrepandum*. There were different bands observed. The FTIR band of 3435 cm−1 (indicating the stretching of aromatic nitrogen-hydrogen bonds), 2922 cm−1 (representing -CH groups), 1590 cm−1 (related to the C=N group), 1398 cm−1 (related to the C-N group), and 629 cm−1(related to the OH− group) (**Figure 5**).

### 3.2 Biological activities

#### 3.2.1 Antimicrobial activity

##### 3.2.1.1 Effect of AgNPs on microbial growth

AgNPs inhibitory impact on *P. aeruginosa* and *F. equiseti* growth was evaluated using the well diffusion method and the PIMG method, respectively. AgNPs exhibited a significant inhibitory effect, as indicated by the formation of a clear zone with a diameter of 21± 0.03 mm, compared to the positive control gentamycin, which had a diameter of 18 ± 0.02 mm against *P. aeruginosa*.

Furthermore, the data demonstrated that AgNPs had an inhibitory effect on the mycelial growth of *F. equiseti*, with a percentage inhibition of 63 ± 0.01% relative to the control.

Gene toxicity of the AgNPs treated microorganisms The impact of AgNPs on the genetic polymorphism of *P. aeruginosa* and *F. equiseti* was assessed by the utilization of ISSR molecular markers. Initially, a total of six ISSR primers were examined, and afterwards, four primers were chosen based on their consistent band reproducibility and the overall number of amplified fragments.

The total number of amplified fragments in *P. aeruginosa* was 19 and exhibiting a polymorphism percentage of 47.5%. In the instance of *F. equiseti*, a total of 16 fragments were successfully amplified, the polymorphism percentage was 45.83% (**Table 1**). **Tables 2** present the comprehensive similarity matrix obtained by ISSR analysis conducted on *P. aeruginosa* and *F. equiseti*.

**Table 1:** ISSR analysis with *P. aeruginosa* and *F. equiseti*.

No.	Primer name	Primer’s sequence	GC%	Tm	*P*. *aeruginosa*			*F. equiseti*		
					Total bands	Polymorphic bands	Polymorphism %	Total bands	Polymorphic bands	Polymorphism %
1	HB-10	GAGAGAGAGAGACC	57.14	44	5	2	20	4	2	50
2	HB-11	GTGTGTGTGTGTCC	57.14	44	4	2	50	6	2	33.3
3	HB-15	GTGGTGGTGGC	72	40	5	4	70	6	4	66.67
4	ISSR-M2	ACCACCACCACCACCACCG	68.42	64	6	3	50	6	2	33.33
Total					20	11	47.5	22	10	45.83

**Table 2:** Total similarity matrix for the antimicrobial effect of AgNPs on the tested microorganisms.

Microorganisms	Groups	Control	AgNPs treated
*F. equiseti*	Control	100	52.9
	AgNPs	52.9	100
*P. aeruginosa*	Control	100	36.3
	AgNPs	36.3	100

#### 3.2.2 Anticancer activity

The cytotoxic properties of AgNPs produced by *S. subrepandum* extract on MCF-7 breast cancer cells were evaluated using the SRB test. The assay was conducted using a range of AgNPs’ dosages, from 0.01 to 100 μg/ml. The study revealed a correlation between cell viability and dosage, as shown in. The cytotoxicity of AgNPs was verified, and the half-maximal inhibitory value (IC_50_) was detected at 12.5 µg/ml.

##### 3.2.2.1 Flow cytometry assay

**Figure 6:**
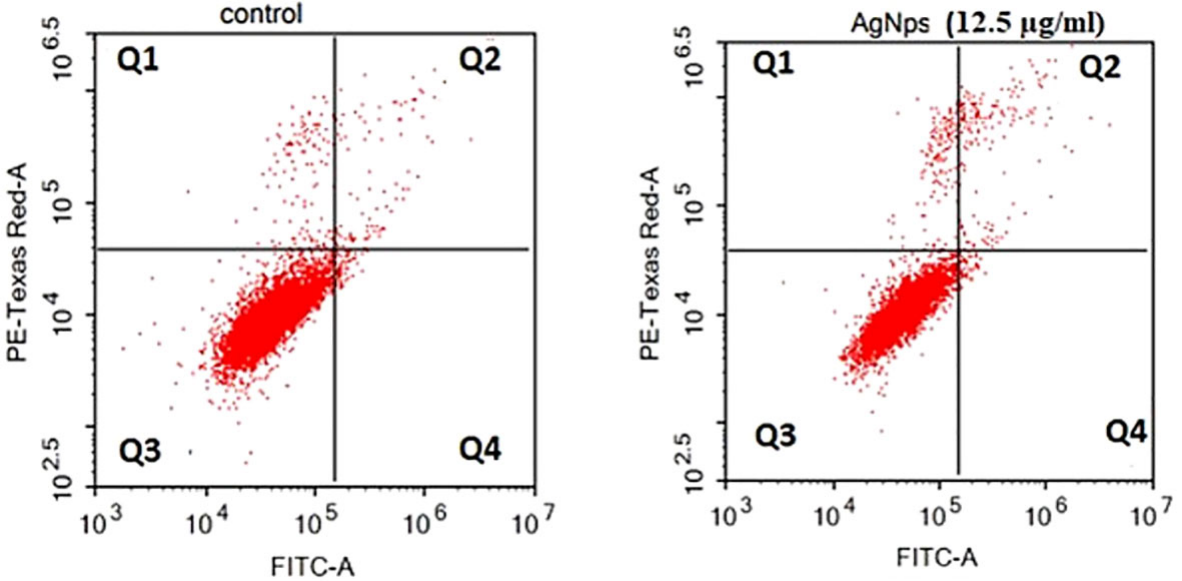
Flow cytometric investigation of AgNPs-induced apoptosis in MCF-7 cell lines after annexin V and PI labelling with FITC. **(A)** MCF-7 control group, **(B)** AgNPs treated group.

The technique of flow cytometry was utilized to quantitatively assess the rates of apoptosis by employing annexin V-FITC/PI labelling. The MCF-7 cells were subjected to treatment with AgNPs at a concentration corresponding to the IC_50_ value (12.5 µg/ml) for 24 h. As depicted in **Figure 6** and **Table 3**, the viability of negative control MCF-7 culture cells was found to be 96.68%. When cells were exposed to AgNPs, the proportion of viable cells drastically decreased at IC_50_ (93.55%). Additionally, compared to the negative control (0.46%), the proportion of cells at an early apoptotic stage significantly increased (0.71%). In addition, there was a substantial rise in the percentage of cells in the late apoptotic stage (3.05%) in comparison to the negative control (1.28%).

**Table 3:** Flow cytometric evaluation of MCF-7 cells following AgNPs treatment.

Groups Phases	MCF-7 control	AgNPs treated (12.5 µg/ml)
**%Viable cells (Q3)**	96.68 ± 0.08*	93.55 ± 0.13^*^
**%Early apoptosis (Q4)**	0.46 ± 0.07a**	0.71 ± 0.13^**^
**%Late apoptosis (Q2)**	1.27 ± 0.11b*	3.05 ± 0.26^*^
**%Necrosis (Q1)**	1.58 ± 0.08b*	2.69 ± 0.19^*^

The values are reported as the average ± SD of three separate studies. * Statistically significant at a significance level of p< 0.05, ** statistically significant at a significance level of p< 0.01 compared to the control group.

The cells were stained with FITC-conjugated annexin V and PI, and thereafter incubated at 37°C for 24 hours.

**Figure 7:**
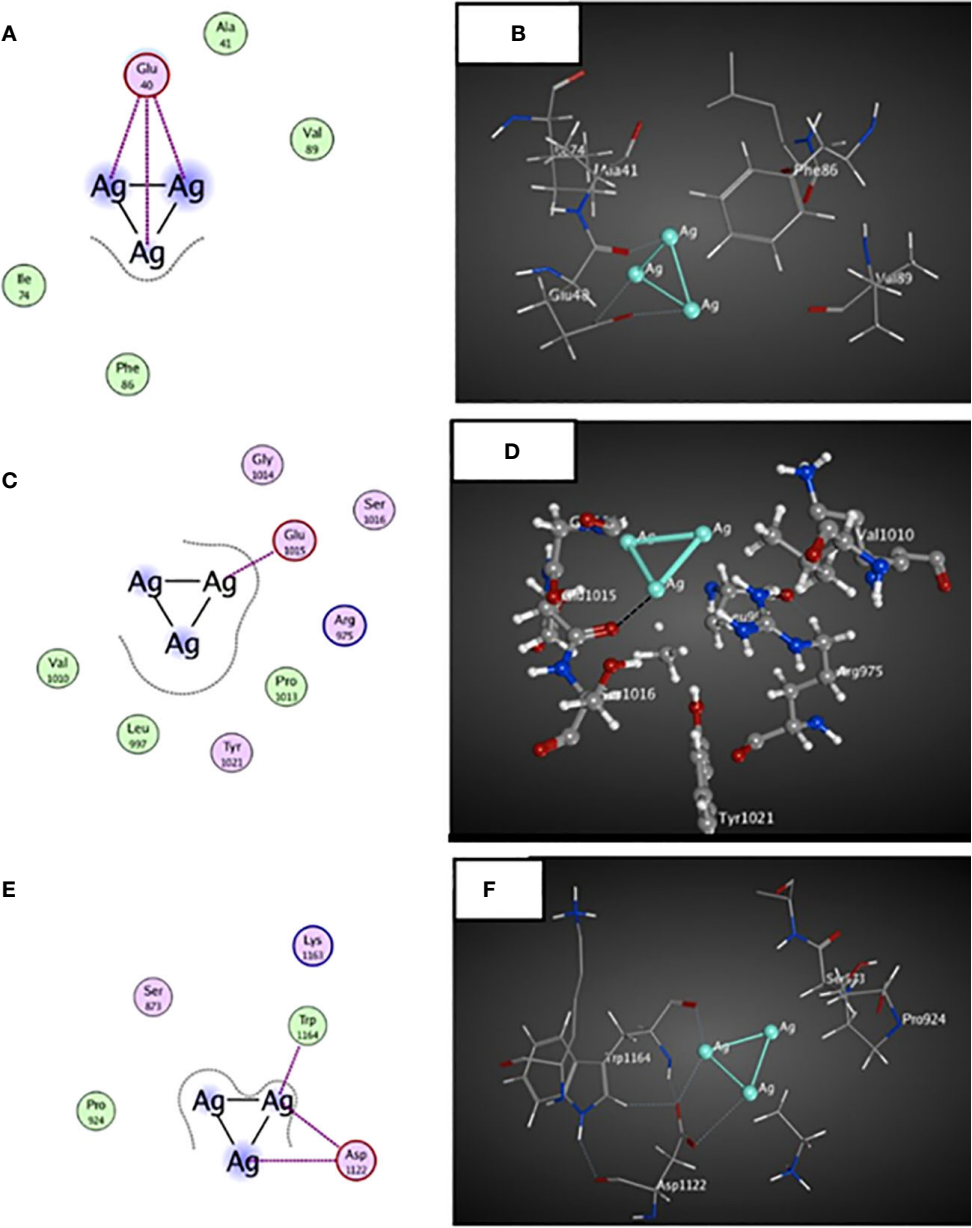
3D and 2D docked interactions map for the ligand molecule Ag atoms with the binding sites found in MCF-7 cells. **(A, B)** represent the 2D and 3D interactions between surviving protein and Ag atoms, **(C,D)** represent the 2D and 3D interactions between JAK2 protein and Ag atoms, **(E,F)** represent the 2D and 3D interactions between BRCA2 proteins and Ag atoms.

A possible interaction occurred between the ligand molecule, Ag atoms, and the survivin protein at the GLU 40 residue (**Figure 7**). Ag atoms demonstrated an inhibitory effect on survivin protein through interaction-free energy. The ability of metal contacts in silico was detected by energy scoring (-3.0, -0.9, -2.4 Kcal/mol) (**Table 4**).

**Table 4:** *In-silico* docking study of surviving protein, JAK2 and BRCA2 proteins involved in breast cell cancer with the ligand Ag atoms.

PDB ID	Docking score (Kcal/mol)	Interaction type	Amino acid residue involved in docking
**Survivin**	-3.0 -0.9 -2.4	Metal	GLU 40
**JAK2**	-1.5	Metal	GLU 1015
**BRCA2**	-0.8 -3.3 -0.5	Metal	ASP 1122 ASP 1122 TRP 1164

The ligand molecule interacted with Ag atoms in the crystal structure of the JAK2 complexed with a strong quinoxaline ATP site inhibitor. The docking results showed that Ag atoms facilitated hydrophobic contact with GLU 1015 residues. Metal interaction ability was detected in-silico using energy score of -1.5 Kcal/mol (**Table 4**).

An interaction occurred between the ligand molecule, Ag atoms, and the crystal structure of a PALB2/BRCA2 complex. The docking results showed that the ligand compound, Ag atoms, interacted with ASP 1122 and TRP 1164 residues (**Figure 7**). The in-silico interaction ability was determined by the metal bonding with docking scores of -0.8, -3.3, and -0.5 Kcal/mol (**Table 4**).

#### 3.2.3 Molluscicidal activity

**Figure 8:**
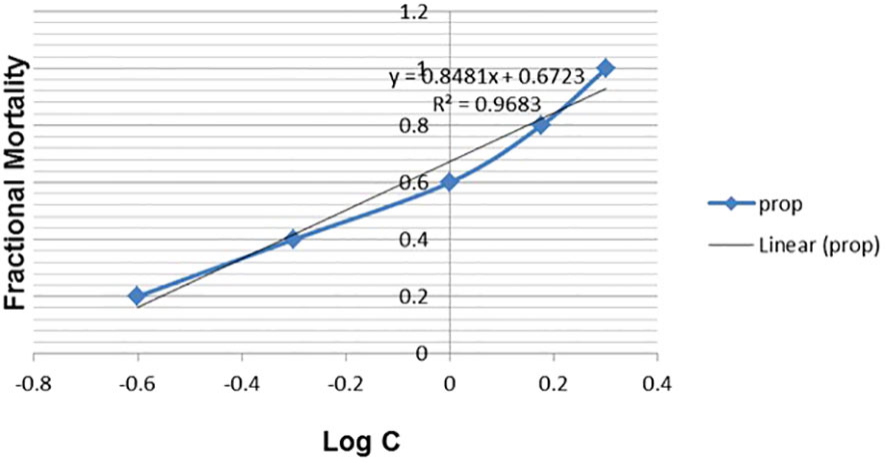
Fractional mortality of *B. alexandrina* snails after 24h exposure to AgNPs.

AgNPs demonstrated significant molluscicidal action on adult *B. alexandrina* snails following a 24-hour exposure period, as indicated in **Table 5**. The half-lethal concentrations (LC_50_) were 0.84 mg/l. The slope value suggested that the probability line had a significant incline and exhibited a strong fit to the curve (**Figure 8**).

**Table 5:** The effectiveness of AgNPs as molluscicidal agent against adult *B. alexandrina* snails after a 24-hour exposure.

Concentration (mg/l)	LC_10_ (mg/l)	LC_25_ (mg/l)	LC_50_ (mg/l)	Confidence interval	LC_90_ (mg/l)	slope
AgNPs treatment	0.11	0.45	0.84	0.47- 1.28	1.58	1.2

**Figure 9:**
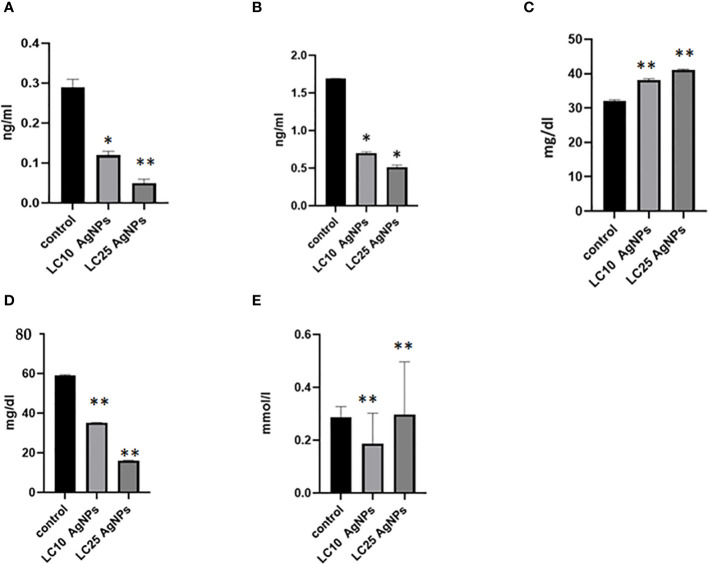
The impact of AgNPs on some biochemical parameters, where **(A)** represents glycogen phosphorylase levels, **(B)** succinate dehydrogenase, **(C)** glucose, **(D)** phospholipids, and **(E)** triglyceride. * Statistically significant at a significance level of p< 0.05; ** statistically significant at a significance level of p< 0.01 when compared to the control group.

The levels of glycogen phosphorylase, succinate dehydrogenase, triglycerides, and phospholipids were significantly decreased after exposing *B. alexandrina* snails to LC_10_ or LC_25_ doses of AgNPs. In contrast, there was a notable increase in glucose levels (p ≤ 0.05) when compared with the control group (**Figure 9**).

After the exposure *B. alexandrina* snails with LC_10_ or LC_25_ AgNPs, a significant decrease in the concentrations of uric acid and creatinine (p ≤ 0.05) on cmparing with the control group (**Table 6**).

**Table 6:** AgNPs’ effect on some kidney function parameters, T and E levels of *B. alexandrina* snails.

parameter Groups	Uric acid (mmol/g)	Creatinine (μmol/l)	Testesterone (nmol/L)	Estrogen (pg/ml)
Control	12.2 ± 0.21	13.5 ± 0.4	19.5 ± 0.3	23.3 ± 0.4
LC_10_ (AgNPs)	7.7± 0.2	10.2 ± 0.2*	12.2 ± 0.2*	15.6 ± 2.1*
LC_25_ (AgNPs)	5.01 ± 0.3*	7.6 ± 0.1**	10.1 ± 0.2**	11.2 ± 1.2**

Following the administration of LC_10_ or LC_25_ of AgNPs to *B. alexandrina* snails, a significant reduction in the concentrations of Testosterone (T) and Estradiol (E) was found (p< 0.05) (**Table 6**).

## 4 Discussion

The current investigation utilized the ethanol extract of S. subrepum for the purpose of biosynthesizing AgNPs. The absorption peak of AgNPs synthesized using *S. subrepandum* was observed at a wavelength of 425 nm. This wavelength corresponded to the 420–480 nm region of AgNPs’ distinctive surface plasmon resonance, according Ærøe Hyllested et al. (2015). The transmission electron microscopy analysis of AgNPs revealed that they are spherical to cube-shaped, have a homogenous distribution, and have an average particle size of 4.5 ± 1.2 nm. The data shown in our study aligns with the findings of Ganapathy Selvam and Sivakumar (2015), who previously reported that the AgNPs synthesized using algal extract exhibited spherical and cuboid shapes, with a particle size ranging from 2 to 55.8 nm. Furthermore, their study indicated that these particles were well distributed, likely due to the influence of electrostatic forces. The FTIR study detected the interaction between the chemical metabolites of *S. subrepum* and AgNPs. The FTIR spectrum of 3435 cm−1 (indicating the stretching of aromatic nitrogen-hydrogen bonds) confirmed the detection of primary amines in algal proteins (Amin et al., 2021). The band at 2922 cm−1 (representing -CH groups) demonstrated the presence of alkyne compound. Simple amines were detected with a wavenumber of 1590 cm−1 that linked with C=N group. The peak at 1398 cm−1 (associated with C-N group) and the peak at 629 cm−1 (associated with the OH− group) were attributed to peptide bond. The data provided evidence of the existence and attachment of proteins to AgNPs, which may result in their potential stabilisation and inhibition of agglomeration. The presence of amino acids and peptides surrounding the silver nanoparticles was confirmed using FTIR spectroscopy. The detection of characteristic peaks of amino acids in the UV–vis spectra confirms the existence of proteins in the cell-free filtrate. Nanoparticle-protein interactions are commonly understood to happen either through free amino groups or cysteine residues found in proteins, or because negatively charged carboxylate groups in enzyme proteins are attracted to one another electrostatically (Mandal et al., 2005). The amino acid residues and peptides exhibited a high affinity for silver due to the presence of carbonyl groups (Balaji et al., 2009). The proteins on the surface of the silver nanoparticles function as capping agents. The findings were consistent with the outcomes obtained using A. niger (Jaidev and Narasimha, 2010; Elamawi et al., 2018).

The current investigation demonstrated that AgNPs exhibited antimicrobial properties and induced genetic changes in *P. aeruginosa* and *F. equiseti*. The results of our study align with previous research conducted by Hayat et al. (2023) and Mouriya et al. (2023), which reveals the antibacterial properties of biosynthetic AgNPs against *P. aeruginosa*. Also, the effect of biogenic AgNPs against *F. equiseti* has been reported (Ahmad et al., 2022; Moond et al., 2023). Multiple processes are involved in the antibacterial action exhibited by AgNPs. One of the mechanisms under consideration involves the impact of AgNPs on microbial DNA. AgNPs exhibit a tendency to adhere to microbial DNA, hence impeding the processes of replication and transcription (Durán et al., 2016). Moreover, the direct contact between AgNPs and DNA can result in structural impairments, fragmentation of DNA strands, and various types of genomic instability, eventually impeding the growth and reproduction of microorganisms (Durán et al., 2016; Tang and Zheng, 2018). Furthermore, it has been documented that AgNPs possess the ability to interact with microbial cell membranes, resulting in structural impairment and alterations in permeability, ultimately compromising the integrity of the membrane (Li et al., 2010). The occurrence of this disturbance can lead to the release of cellular components, such as ions and proteins, ultimately resulting in cellular death (Li et al., 2010; Vazquez-Muñoz et al., 2019). Also, AgNPs have the ability to stimulate the production of reactive oxygen species (ROS) within microbial cells. ROS induce oxidative stress, hence leading to the impairment of cellular constituents such as proteins, lipids, and nucleic acids (Nisar et al., 2019). The oxidative damage has a significant role in the overall antibacterial efficacy of AgNPs. Additionally, AgNPs could bind with microbial proteins, leading to disruption of their structure and function. This disruption can even extend to essential enzymes involved in the respiratory chain, which is achieved by destroying the plasma membranes of the microorganisms (Nisar et al., 2019). The antibacterial properties of silver nanoparticles generated by algae have been documented to be effective against Staphylococcus species and Proteus mirabilis, as reported by Hamouda and Aljohani (2024) and Rose et al. (2024).

This study examined the anti-cancer effects of phyco-synthesized AgNPs against the MCF-7 breast cancer cell line. The data that was found from the SRB experiment demonstrated a dose-dependent cytotoxic effect of AgNPs on the cancer cells, with an IC_50_ value of 12.5 µg/ml. Flow cytometry further explores apoptosis and necrosis in treated cells, enhancing the understanding of the AgNPs’ mode of action. AgNPs have been shown in earlier research to exhibit anticancer effects on a number of cancer cell lines, including MCF-7 and MDA-MB-231 for breast cancer, SiHa for cervical cancer, and A549 for lung cancer (Algotiml et al., 2022; Salve et al., 2022; Manimaran et al., 2023). More over, the anticancer propertise of AgNPs biosynthesized using algea has been documented (Rose et al., 2024). The potential mechanisms underlying the cytotoxic effects of AgNPs on cancer cells may involve the induction of oxidative stress. Earlier investigations have suggested that AgNPs have the capacity to induce oxidative stress in various types of cancer cells (Manimaran et al., 2023). AgNPs possess a tiny size and exhibit strong surface reactivity, which enables them for inducion of reactive oxygen species (ROS). Consequently, this ROS production can result in oxidative damage to several cellular constituents (Manimaran et al., 2023). The correlation between the rise in oxidative stress and the observed apoptotic and cytotoxic effects on cancer cells subsequent to AgNPs therapy has been reported (Barcińska et al., 2018). Another potential mechanism of cytotoxicity is the direct contact between silver nanoparticles (AgNPs) and the cellular membrane. AgNPs have the potential to disturb the cell membrane, resulting in increased permeability and compromised integrity. This may lead to the leakage of cellular contents, loss of ion homeostasis, and ultimately cell mortality (Sun et al., 2016).

Molecular docking can be utilised as a method to elucidate and forecast the behaviour of ligand Ag atoms against specific proteins through receptor-ligand interactions.Human breast cancer cells can evade apoptotic cell death through various mechanisms. Therapies often lose effectiveness when inhibitor of apoptotic proteins (IAP) medicines are overexpressed. Survivin, as part of the IAP protein family, may play a role in inhibiting caspases, which are involved in apoptosis, according to Mahdavi et al. (2016). The current findings demonstrated an interaction with the ligand, Ag atoms, and survivin proteins, which could potentially result in the suppression of survivin protein.The survival and dissemination of breast cancer cells rely on the activation of JAK2, which establishes the Lnc-BM/JAK2/STAT3 signalling pathway (Abu Bakar et al., 2018). The molecular docking of JAK2 with Ag atoms indicated a possible interaction that could reduce the involvement of JAK2 in breast cancer cells. BRCA2 plays a crucial role in DNA damage repair by homologous recombination (Nguyen et al., 2022). The molecular docking revealed a possible contact between silver atoms and BRCA 2 peptide, suggesting a potential reduction in the effects of BRCA2 mutations.

The study investigated the molluscicidal properties of AgNPs synthesized by the ethanol extract of *S. subrepum* against adult *B. alexandrina* snails, demonstrating a significant impact on snail mortality. Biochemical parameters and hormone levels in the snails are assessed, adding a layer of complexity to the study by considering broader ecological implications beyond direct toxicity.

A particular enzyme called glycogen phosphorylase catalyzes the initial, regulated phase of glycogen breakdown to produce glucose 1-phosphate (Rocha Leão, 2003). The most significant anaerobic energy source for animal tissues is glucose, which is stored in reserve as glycogen in the liver and muscles (Migocka-Patrzałek and Elias, 2021). The present study indicated a significant decrease in glycogen phosphorylase and succinate dehydrogenase, while glucose levels were significantly increased when *B. alexandrina* snails were exposed to LC_50_ AgNPs. The amount of available glycogen decreases as a result of the snail having to accelerate glycolysis as it attempts to fulfil its energy demands. Glucose levels may serve as a reliable signal in experiments monitoring environmental stresses. The increase in glucose levels may be compensated with these decreases in glycogen phosphorylase (adel Abdel-Khalek et al., 2015).

Triglycerides are largely hydrolyzed into free fatty acids, which are subsequently oxidized in the mitochondrial matrix to produce the energy needed to meet metabolic demands and reduce the negative consequences of any stressors (Hassan et al., 2021). Phospholipids (PLs) are present in the membranes of both plant and animal cells. These amphiphilic lipids are arranged in a lipid bilayer structure (Penagos-Tabares et al., 2018). Triglycerides and phospholipids were significantly decreased when *B. alexandrina* snails were exposed to LC_50_ AgNPs. The primary factors influencing changes in lipid profiles include stressful circumstances and environmental hazards (Assadi, 2017). Lustrino et al. (2010) concluded that stress causes snails to become less active and have a lower probability of finding food, which leads to an increase in the use of carbohydrate reserves and triglycerides as an alternative energy substrate, which leads to a fall in their levels.

Endocrine systems in invertebrates often regulate processes like growth, development, and reproduction. The disruption of endocrine system with any environmental or chemical factors may modify the hormonal balance and gametogenic processes (Ibrahim et al., 2023). The present study demonstrated a significant drop in bo th testosterone (T) and estrogen (E) levels in *B. alexandrina* snails after being exposed to LC_50_ AgNPs, as compared to the control group. Similarly, Ibrahim and Sayed (2019) reported that the levels of both testosterone (T) and oestrogen (E) dropped following exposure to sublethal doses of oxyfluorfen 24%EC. Steroid hormone fluctuations may cause the snail’s population to decline as well as its fertility.

The current finding demonstrated a significant decrease in creatinine and uric acid levels following *B. alexandrina* snail’s exposure to LC_50_ AgNPs. Creatinine is thought to be a waste product produced in the muscle from creatinine phosphate, a substance with a high energy storage (Ziętek et al., 2022). Numerous animals required uric acid in order to properly excrete nitrogen. Uric acid was later suggested to have additional physiological functions, including those of an antioxidant and a source of combined carbon and nitrogen that can be regenerated into proteins (Giraud-Billoud et al., 2011).

## 5 Conclusions

Current research highlights the utilization of brown macroalgae as a sustainable and environmentally friendly source for nanoparticle production. The comprehensive analysis indicates that the silver nanoparticles synthesized by algae have potent antibacterial, anticancer, and molluscicidal activities. Including *in silico* molecular docking research offers insight into the interaction between AgNPs and target biomolecules, showcasing the promise of algal-synthesized AgNPs for medical and environmental applications. This integration of practical experimentation and computational simulation represents a significant progress in nano-biotechnological applications, paving the way for future research on using marine resources to produce nanoparticles with various biological properties. Future research should thoroughly investigate the wide range of potential uses for silver nanoparticles created from macroalgae, increase efforts to improve production efficiency, and carefully monitor their long-term effects on the environment and living organisms. This comprehensive strategy will not only expand the range of applications for these nanoparticles, but also guarantee their long-term incorporation into other sectors.

## Data availability statement

The datasets presented in this study can be found in online repositories. The names of the repository/repositories and accession number(s) can be found in the article/supplementary material.

## Author contributions

HE: Conceptualization, Data curation, Formal analysis, Funding acquisition, Investigation, Methodology, Project administration, Resources, Software, Supervision, Validation, Visualization, Writing – original draft, Writing – review & editing. AA: Conceptualization, Data curation, Formal analysis, Funding acquisition, Investigation, Methodology, Project administration, Resources, Software, Supervision, Validation, Visualization, Writing – original draft, Writing – review & editing. MM: Conceptualization, Data curation, Formal analysis, Funding acquisition, Investigation, Methodology, Project administration, Resources, Software, Supervision, Validation, Visualization, Writing – original draft, Writing – review & editing. HS: Funding acquisition, Writing – review & editing. AI: Methodology, Writing – original draft. ET: Supervision, Validation, Visualization, Writing – original draft, Writing – review & editing, Conceptualization, Data curation, Formal analysis, Funding acquisition, Investigation, Methodology, Project administration, Resources, Software.
